# Oxidative stress, activity behaviour and body mass in captive parrots

**DOI:** 10.1093/conphys/cov045

**Published:** 2015-10-20

**Authors:** S D Larcombe, C A Tregaskes, J Coffey, A E Stevenson, L G Alexander, K E Arnold

**Affiliations:** af1 Institute of Biodiversity, Animal Health and Comparative Medicine, University of Glasgow, Glasgow G12 8QQ, UK; af2 WALTHAM® Centre for Pet Nutrition, Waltham-on-the-Wolds, Leicestershire LE14 4RT, UK; af3 Environment Department, University of York, Heslington, York YO10 5DD, UK

**Keywords:** Antioxidants, birds, budgerigars, carotenoids, comet assay, malondialdehyde

## Abstract

Many parrot species are kept in captivity for conservation, but often show poor reproduction, health and survival. These traits are known to be influenced by oxidative stress, the imbalance between the production of reactive oxygen species (ROS) and ability of antioxidant defences to ameliorate ROS damage. In humans, oxidative stress is linked with obesity, lack of exercise and poor nutrition, all of which are common in captive animals. Here, we tested whether small parrots (budgerigars, *Melopsittacus undulatus*) maintained in typical pet cages and on *ad libitum* food varied in oxidative profile, behaviour and body mass. Importantly, as with many birds held in captivity, they did not have enough space to engage in extensive free flight. Four types of oxidative damage, single-stranded DNA breaks (low-pH comet assay), alkali-labile sites in DNA (high-pH comet assay), sensitivity of DNA to ROS (H_2_O_2_-treated comet assay) and malondialdehyde (a byproduct of lipid peroxidation), were uncorrelated with each other and with plasma concentrations of dietary antioxidants. Without strenuous exercise over 28 days in a relatively small cage, more naturally ‘active’ individuals had more single-stranded DNA breaks than sedentary birds. High body mass at the start or end of the experiment, coupled with substantial mass gain, were all associated with raised sensitivity of DNA to ROS. Thus, high body mass in these captive birds was associated with oxidative damage. These birds were not lacking dietary antioxidants, because final body mass was positively related to plasma levels of retinol, zeaxanthin and α-tocopherol. Individuals varied widely in activity levels, feeding behaviour, mass gain and oxidative profile despite standardized living conditions. DNA damage is often associated with poor immunocompetence, low fertility and faster ageing. Thus, we have candidate mechanisms for the limited lifespan and fecundity common to many birds kept for conservation purposes.

## Teaser

Captive breeding programmes are common for many parrot species. We show that in small parrots kept in standard cages with ad libitum food, high weight gain was prevalent and linked with a risk of DNA damage. Such damage could explain the poor health, fertility and survival of many captive parrots.

## Introduction

Members of the Psittaciformes order (parrots) are particularly endangered and also long lived among bird species (Forshaw, 2010; Young *et al.*, 2012). In response to declines in population sizes, many parrot species are kept in captivity for conservation purposes (White *et al.*, 2012; Young *et al.*, 2012). For example, for the critically endangered Puerto Rican parrot (*Amazona vitatta*) a captive breeding programme was established in 1973 from a mere 13 birds (Earnhardt *et al.*, 2014). Despite problems with low fertility and inbreeding (Snyder *et al.*, 1996), this aviary population now supplies an actively managed, long-term reintroduction programme (Earnhardt *et al.*, 2014). Recent research using zoo records revealed that while some individual parrots in captivity can live to great ages (>20 years in some cases), the median maximal lifespan is usually <30% of this duration for most species (Young *et al.*, 2012). Advances in animal husbandry seem to be resulting in greater longevity for parrots in zoos today compared with those over a decade ago, but clearly many parrot species are not thriving in captivity (Young *et al.*, 2012). Likewise, breeding success can be relatively low among captive birds, in particular captive-bred birds (Snyder *et al.*, 1996), with a number of causal factors implicated, such as low sperm counts, low sperm or egg quality and embryo mortality (Houston *et al.*, 2007; Hemmings *et al.*, 2012; Young *et al.*, 2012). Obesity, lack of exercise and poor nutrition have all been suggested to impact upon reproductive performance, health and survival in many taxa and are all common in captive, companion and managed animals (Houston *et al.*, 2007; Clubb *et al.*, 2009; German *et al.*, 2012). Given that oxidative stress can influence many components of the life history (reviewed by Monaghan *et al.*, 2009), it might provide an underlying mechanism linking many of the problems found in captive birds.

There is growing evidence that a number of life-history traits, including fertility, immune function, sexual attractiveness, fecundity and longevity, are modulated by oxidative stress (reviewed by Monaghan *et al.*, 2009; Selman *et al.*, 2012). Indeed, some have suggested that oxidative stress is a key cellular mechanism that constrains both lifespan and life-history strategies (Costantini *et al.*, 2013; Metcalfe and Monaghan, 2013). Reactive oxygen species (ROS) are unstable molecules, produced by cell-signalling processes, by the immune system and during metabolism (Murphy *et al.*, 2011). Although the production of some ROS is natural and unavoidable, unchecked ROS cause damage to the lipids, proteins and DNA necessary for maintaining biological function (Knight, 1998). This naturally produced oxidative stress has led to the evolution of endogenous antioxidant defences within all animals (Surai, 2002). Some antioxidants are also acquired through the diet, which might augment the antioxidant defences of animals (e.g. Surai, 2002; Kolosova *et al.*, 2006), including birds (e.g. Woodall *et al.*, 1996; Larcombe *et al.*, 2008; but see Costantini and Møller, 2008). Oxidative stress occurs when the body's antioxidant systems are overwhelmed by the production of ROS. Although as yet poorly understood (Monaghan *et al.*, 2009), the factors affecting an individual's susceptibility to ROS and the potential impacts of ROS on health, fertility and survival are therefore of relevance to conservation physiologists. There are several mechanisms by which captive bird species might be particularly vulnerable to oxidative stress, and this could have important implications for conservation. In the present study, we investigate the following: (i) the propensity to reach suboptimal body mass with *ad libitum* food provision; and (ii) reduced ability for exercise training.

One of the internationally recognized five freedoms for animal welfare in captive animals is ‘freedom, from hunger, thirst or malnutrition’ (OIE, 2014). In practice, this means that many captive animals are provided with an *ad libitum* diet, with the assumption that where food requirements are unknown (as is generally the case with captive birds; Koutsos *et al.*, 2001; Kalmar *et al.*, 2010), animals are a better judge of their requirements than keepers (Fidgett and Gardner, 2014) This *ad libitum* diet, combined with a restriction on or non-requirement for natural exercise and potential preferences for food items that would be restricted in wild environments, means that captive birds, especially parrots (Kalmar *et al.*, 2010), may consume more food than required, reaching suboptimally high body mass (Fidgett and Gardner, 2014). In wild birds, high body mass is generally considered to enhance fitness, but in captivity it can potentially signal ‘obesity’, with concomitant health and performance problems (Larcombe *et al.*, 2008). Obesity, or suboptimally high body mass, has been repeatedly connected with oxidative stress and damage, both because it may accelerate the production of ROS (Aroor and DeMarco, 2014) and because oxidative stress in an excess of body fat is an important mechanism for pathogenic syndromes related to high body mass (Furukawa *et al.*, 2004).

Here, using captive budgerigars (*Melopsittacus undulatus*) as a model for captive psittaciforme birds, we test the hypothesis that increased body mass, associated with access to *ad libitum* food in captivity, will result in increased oxidative damage. While we acknowledge that the budgerigar is smaller and has a more granivorous diet than many other parrots, we feel that it is a useful model of psittaciformes because it shares many of the behavioural and physiological traits typical of parrots, such as monogamous pair breeding, relatively long lifespan and relatively high intelligence (Brockway, 1964; Harper, 1998; Kalmar *et al.*, 2010).

A second factor relating to both welfare and oxidative stress in captive animals relates to the issue of physical exercise. Given that ROS production is increased by metabolic processes, an individual's level of physical activity is likely to alter its oxidative balance (Urso and Clarkson, 2003). Several studies have shown that exercise, particularly strenuous exercise, can increase oxidative stress in humans and other animals (Hartmann *et al.*, 1995; Aniagu *et al.*, 2006), including birds (Costantini *et al.*, 2007). In apparent contrast, we have previously shown that exercise-linked oxidative damage, in the form of lipid peroxidation, was ameliorated in captive budgerigars after they had been subjected to regular exercise sessions during which they were trained to perform take-off escape flights, a strenuous and biologically relevant form of exercise. However, even though guidelines for maintaining birds in captivity generally recommend cages that provide for enough space to spread the wings, hop around the cage or even allow short flights, many cages do not permit extensive aerobic exercise. Thus, such cages may limit the capacity of the birds within to develop resistance to exercise-generated oxidative stress, resulting in oxidative damage caused by high activity levels. To date, studies of oxidative balance and exercise in animals usually involve enforced strenuous exercise, but the extent to which an individual's ‘natural behaviour’ will affect levels of oxidative stress is unclear. Here, we test the prediction that birds that are generally more active in their cages will have increased levels of oxidative damage than more sedentary animals, if attenuation of exercise-mediated oxidative damage does not occur.

In the present study, we explored whether the oxidative profile in adult budgerigars fed a standard *ad libitum* diet was related to activity levels or body mass. To account for possible confounding effects of a captive diet, i.e. insufficient quantities of dietary antioxidants or avoidance of antioxidant-rich food items, we also measured food choice, food intake and plasma antioxidant levels. To investigate the implications of oxidative stress, we assessed it via direct quantification of oxidation products of lipids and DNA; measurement of malondialdehyde (MDA), a product of lipid peroxidation, is one of the most commonly used techniques (Young and Trimble, 1991). We also gauged DNA damage because it is estimated that ROS are responsible for ∼10 000 base modifications a day (Diplock, 1994). The single-cell gel electrophoresis or comet assay measures DNA fragmentation after electrophoresis to assess the level of DNA damage (Tice *et al.*, 2000). Comet assay can be applied at different alkalinities in order to reveal different types of DNA damage. At a lower pH, it is thought to reveal only DNA strand breaks, and at higher pH both DNA strand breaks and alkali-labile sites are revealed. The yield of DNA breaks after exposure of intact cells to ROS via treatment with hydrogen peroxide (H_2_O_2_) indicates changes in the sensitivity of the cells towards exogenous oxidative DNA damage and thus gives information on antioxidant defences (reviewed by Hoelzl *et al.*, 2009). The comet assay technique may be particularly useful in birds, from which nucleated blood cells can be obtained easily with little impact. Studies on birds, except for those on commercial poultry, have only started to use comet assays and MDA assays in the last few years (e.g. Larcombe *et al.*, 2008, 2010b; Bonisoli-Alquati *et al.*, 2010; Hegseth *et al.*, 2011; Sepp *et al.*, 2012). Our specific aims were to determine whether, in captive budgerigars fed *ad libitum* food and held in cages that met UK governmental guidelines for welfare but did not allow for vigorous exercise, oxidative profile and plasma concentrations of dietary antioxidants were associated with (i) variation in body mass or (ii) differences in activity profiles.

## Materials and methods

Domesticated (green and yellow) budgerigars, 12 male and 12 female, had been maintained in large mixed-sex flight aviaries that permitted free flight over large distances (>2 m high and >10 m long). These birds had been captive reared in the UK. At the start of the experiment, they were weighed and their health status checked by the resident veterinarian. Birds were then randomly housed with a member of the opposite sex because we wished to reduce the possible influence of physiological stress on our results, and evidence suggests that budgerigars are most content in mixed-sex pairs. The budgerigars did not breed during the experiment, and environmental conditions (temperature and lighting regimen) were held constant from their previous cages to prevent opportunistic breeding. We did not observe courtship behaviour or any other indications that birds prepared for breeding during the course of the experiment, and after the experiment the birds were monitored for a further 2 months, in which breeding did not occur. Each pair was housed in a cage measuring 1002 mm × 545 mm × 410 mm, which while too small for free flight was larger than a standard pet breeding cage for this species and met UK Home Office guidelines for captive laboratory bird welfare (Hawkins *et al.*, 2001). Birds had *ad libitum* access to water and food throughout the experiment, except during food-choice trials. Video-monitoring equipment was placed in front of each cage from the start of the study to habituate the birds to it prior to the behaviour trials.

This experiment lasted 28 days, with all budgerigars receiving the same diet of standard Trill^®^, which consists of a seed mix with 3% inclusion of Nutrivit^®^. They also received this diet prior to the start of the trial in their flight aviaries and so were accustomed to it. Nutrivit^®^ is a vitamin supplement in the form of a small seed-like grain that is mixed into the seed mix (Mars, Csongrad, Hungary) and provides a higher concentration of antioxidants, calcium and iodine than is present in seed alone, thus supplying budgerigars with a nutritionally enhanced diet (Brue, 1994). In providing a diet rich in antioxidants, we wished to omit the possibility that the results of our measures of oxidative damage were not a byproduct of an antioxidant-poor diet. Concentrations of antioxidants in the seed diet were as follows: α-tocopherol, 0.75 IU/g; retinol, 0 IU/g; vitamin C, 1.35 µg/g; β-carotene, 0.06 µg/g; lutein, 4.19 µg/g; and zeaxanthin, 1.35 µg/g. Concentrations of antioxidants in Nutrivit^®^ were as follows: α-tocopherol, 1668.4 IU/g; retinol, 220 000 IU/g; vitamin C, 764.4 µg/g; β-carotene, 0.6 µg/g; lutein, 2.8 µg/g; and zeaxanthin, 2.6 µg/g (full nutritional analysis by Eurofins, Wolverhampton, UK). From day 24 to 28, behavioural and diet-choice trials were performed.

All birds were blood sampled after 28 days of the experiment. In order to comply with ethical standards, samples were not collected at the start of the experiment as well. Tarsus, wing and mass measurements were taken. Change in body mass was calculated as the body mass after 28 days minus the body mass at the start of the experiment. A small blood sample (∼250 µl) was taken from the jugular vein via a 25-gauge needle and a syringe. Fifty microlitres of the whole blood was diluted in 1 ml of phosphate-buffered saline immediately in a sodium citrate tube for comet assay. All individuals were subjected to the same capture, restraint and sampling protocols. We have previously shown that measures of oxidative damage and antioxidant defences are not significantly affected by the time between capture and blood sampling (Arnold *et al.*, 2015). Capillary tubes of blood were centrifuged for 5 min at 14 000***g***, and plasma was stored at −70°C, prior to antioxidant and MDA analysis.

### Food intake and behavioural activity

We monitored food intake and behaviour simultaneously at the end of the trial, when birds were most likely to be acclimated to their environment and thus displaying normal patterns of food choice and behaviour. We weighed seed in and out of the cages, and video recorded birds to assess their levels of activity in order to relate this to their oxidative profiles (see also Supplementary Materials and Methods). At 08.00 h on days 24–26 of the experiment, feeding dishes were removed from each cage for a period of 2 h to standardize hunger for food-intake trials, and cages were cleaned. Next, pairs were separated with a cage divider for the duration of the observations. Individual budgerigars were presented with a food bowl containing a prepared 10 g food sample comprising identical proportions of each seed and Nutrivit^®^. The video camera in front of each cage was switched on during the food-choice trial to record behaviour without the confounding effects of social interactions, but the birds were not acoustically isolated. The dish and tray, along with any spilled seed, were removed after 2 h. The remaining seeds were carefully weighed, and Nutrivit^®^ pieces were counted, to monitor food intake as well as potential selection of antioxidant-rich food items. Initial analyses showed that the first hour of each observation period accurately reflected the behaviour of the budgerigars over the entire filmed period. Thus, for each individual, we scored the frequency of different behaviours performed per 10 min interval during the first hour of the trial and averaged across 3 days (see Supplementary Materials and Methods for full description of behaviours). The mean behavioural profiles of birds across the three 1 h observation periods are shown in the Supplementary Results. The birds performed many different behaviours that may be considered ‘active’, and not all individuals performed each of them (e.g. some would hop between perches, while others flew). Behaviours we counted as active were walking, flying, hopping, climbing and turning. In order to reduce the complexity of the behavioural activity, and to avoid multiple comparisons, we used principal component analysis (PCA; SPSS) to create new variables based on these active behaviours that explained the variance in these data. The PCA created two new activity scores, namely Activity PC1 (explaining 43% variance in behavioural scores) and Activity PC2 (explaining 29% variance in behavioural scores). Loading on PC1 was explained mostly by hopping, walking and flying (eigenvalues 0.885, 0.868 and 0.466, respectively) and PC2 was explained mostly by flying, climbing and turning (eigenvalues 0.821, 0.762 and 0.701, respectively).

### Analysis of malondialdehyde

The MDA method was based on that of Young and Trimble (1991; see the Supplementary Materials and Methods and Larcombe *et al.*, 2008 for full details). Briefly, following extraction, the supernatant was analysed on a Summit HPLC system (Dionex, Idstein, Germany) using Chromeleon software (Dionex). An Acclaim 120 C18 5 (4.6 mm × 250 mm column; Dionex) and guard were used with fluorescence detection (excitation, 532 nm and emission, 553 nm). The mobile phase was isocratic, 40:60 methanol:phosphate buffer (40 mM, pH 6.5), with a flow rate of 1 ml/min and a run time of 7 min. Samples were assayed against a standard of malonaldehyde bis (dimethyl acetal; Sigma Aldrich, Poole, UK) that was taken simultaneously through the same procedure.

### Comet assay

For each bird, the following three different treatment regimens were used: carrying out the electrophoresis at two different pH values (high, ∼pH 13.5 and lower, ∼pH 12.5), and in addition, we treated cells with H_2_O_2_ at a lower pH. High pH reveals both DNA single-strand breaks and alkali-sensitive sites, whereas the lower pH reveals only DNA single-strand breaks. Exposure to H_2_O_2_ is believed only to cause breaks, but not at alkali-sensitive sites, and is suggested to indicate the susceptibility of DNA to oxidative damage. Hydrogen peroxide is a natural source of oxidative damage in cells, causing a spectrum of DNA lesions, including single- and double-strand breaks (reviewed by Hoelzl *et al.*, 2009). The comet assay involved slow-spin preparation of avian lymphocytes, treatment of cells with H_2_O_2_ and embedding in agarose-coated slides, following the procedure of Tice *et al.* (2000). Next, we performed electrophoresis at low pH (0.03 M NaOH) to reveal DNA strand breaks and electrophoresis at high pH (0.3 M NaOH), which also converts alkali-labile sites into single-strand breaks. Slides were made and analysed on the same day as blood sampling. Full details of the methods are in the Supplementary Materials and Methods and Larcombe *et al.* (2008). The slide was viewed by epifluorescence microscopy using an Olympus BX-51 (Olympus Optical Co., Tokyo, Japan) with a 460 nm ultraviolet filter for SYBR Green. Komet software (v.6, Kinetics Imaging, Nottingham, UK) was used for image analysis on 100 randomly selected cells for each bird and pH treatment. Cells were scored according to the percentage of DNA in the comet head, as a measure of DNA intactness. The mean intactness was calculated across the 100 cells per slide and across the two slides per treatment per bird. There was high repeatability (>80% across the two slides per treatment per bird). This was then converted to the percentage of damaged DNA to aid interpretation of the results.

### Plasma antioxidants

We analysed levels of α-tocopherol, lutein, zeaxanthin and retinol in order to uncover any effect of oxidative damage, body mass or activity on plasma antioxidant profile. See Supplementary Materials and Methods and Larcombe *et al.* (2008) for further details. Following the extraction process, a Spectra Model 8800 HPLC pump system with a Phenomenex 250 mm × 2 mm i.d. column was used to determine the antioxidant composition of each sample. Using a Diode array absorbance detector type Thermo model UV6000, we detected carotenoids by absorbance at 445 nm, α-tocopherol at 295 nm and retinol at 325 nm. Peaks were identified by comparison with chromatography and retention times of several standards (Sigma, Poole, UK; Fluka, Gillingham, UK).

### Statistics

To test our two main aims of relating oxidative stress to body mass and activity, for each measure of oxidative damage we constructed a general linear model (GLM; SPSS version 20). Oxidative damage measures (4 × comet assays and MDA) were entered as explanatory variables, with the following covariates: mass at start; change in body mass; activity PC1; and activity PC2. The proportion of intact DNA was subtracted from 1 to give the proportion of damaged DNA, and was then arcsine square root transformed prior to analysis. Count data were square root transformed prior to analysis to meet the assumptions of the models. Neither age nor sex significantly explained variance in our data, and they were therefore omitted. Additionally, to test for relationships between feeding duration and body mass measures and between antioxidants and oxidative stress, we used Spearman's rho, because not all the variables met the assumptions of parametric correlations even after transformation. Given the multiple comparisons between all of these measures, we used Bonferroni correction for our *P*-values. Non-significant terms were removed from the model in a backwards stepwise fashion.

### Ethical note

All work was carried out in accordance with the guidelines of the Association for the Study of Animal Behaviour/Animal Behavior Society for the treatment of animals in research and subjected to ethical review by WALTHAM^®^ Centre for Pet Nutrition and the University of Glasgow. No birds became ill or died during this experiment.

## Results

### Analysis of malondialdehyde and comet assay

There was inter-individual variation in all four measures of oxidative damage, as follows: MDA (range 0.068–0.602 μM/l, mean 0.24 ± 0.02 μM/l); percentage of damaged DNA with high-pH comet assay (range 7.18–60.27%, mean 32.17 ± 2.64%); percentage of damaged DNA with low-pH comet assay (range 7.91–46.32%, mean 20.19 ± 2.62), and percentage of damaged DNA with H_2_O_2_-treated comet assay (range 24.41–76.42%, mean 62.28 ± 2.66%).

**Figure 1:**
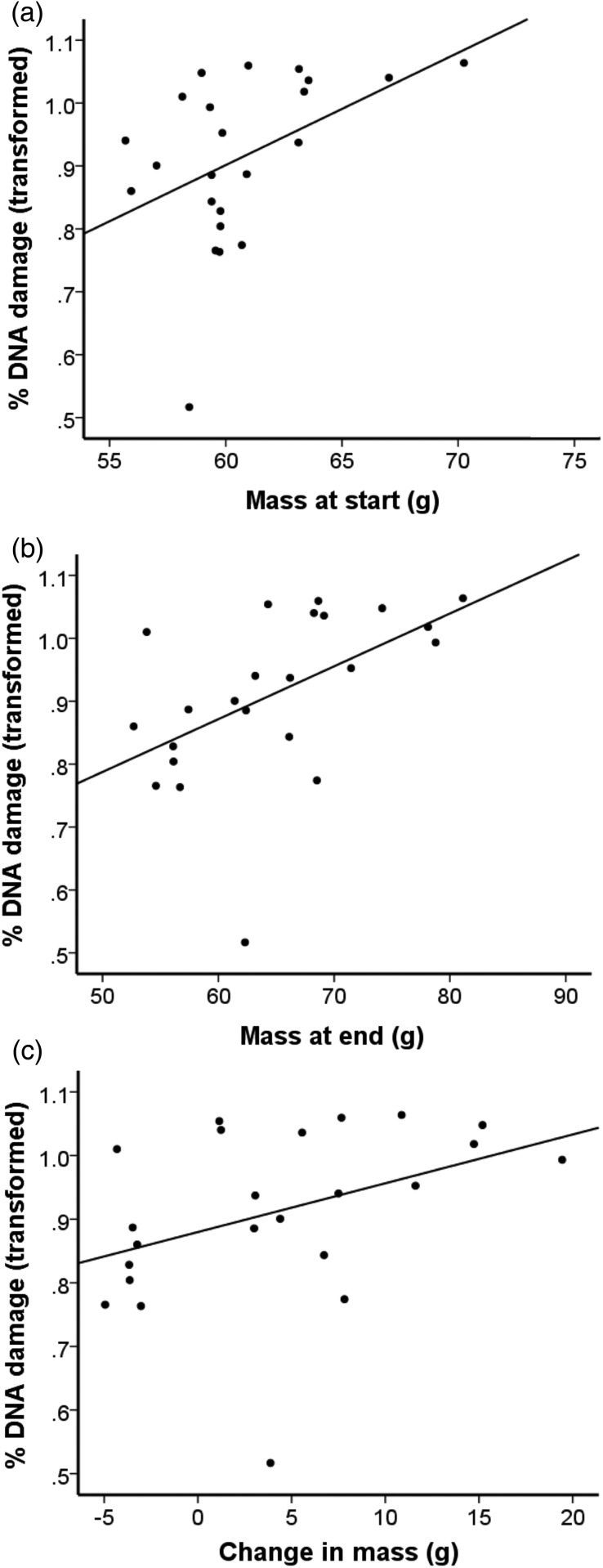
Proportion of damaged DNA measured using the H_2_O_2_-treated comet assay (transformed) and body mass at the start of the experiment (**a**), body mass at the end of the experiment (**b**) and change in body mass (**c**).

The proportion of damaged DNA following H_2_O_2_ treatment was almost significantly related to body mass at the start (GLM *F* = 4.182, d.f. = 1,22, *P* = 0.054; Fig. 1a) and significantly correlated with body mass at the end (GLM *F* = 7.7, d.f. = 1,22, *P* = 0.011; Fig. 1b). A similar, but statistically non-significant, pattern was shown for the change in body mass during 28 days in a small cage and the proportion of damaged DNA following H_2_O_2_ treatment (GLM *F* = 3.09, 1,22, *P* = 0.094; Fig. 1c). Thus, birds that were heavier at the start or end of the experiment had DNA that showed more sensitivity to ROS damage than lighter individuals, as did those that gained most weight during the course of the trial. Body mass measures were uncorrelated with MDA, high-pH and low-pH comet assays.

**Figure 2:**
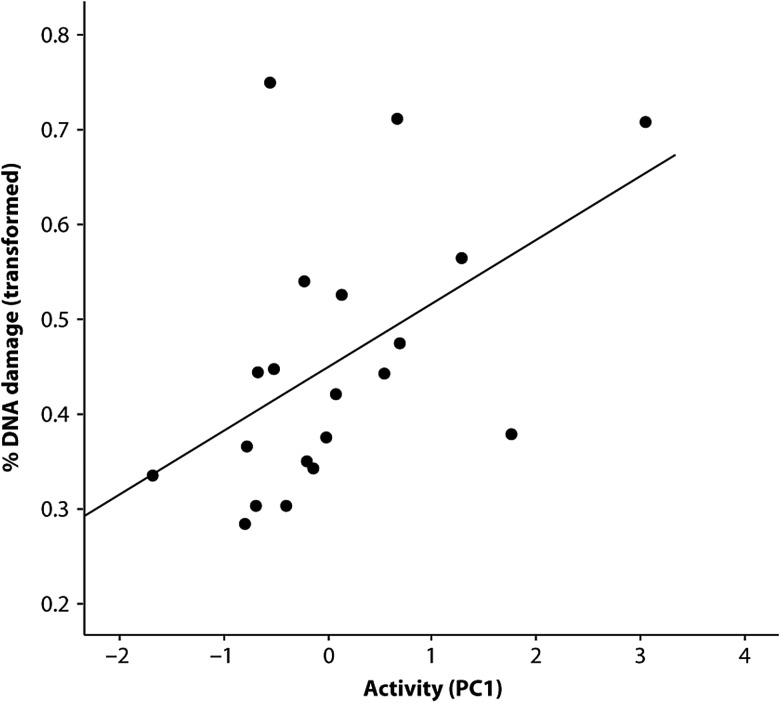
More active birds had significantly higher levels of damaged DNA (single-strand breaks), measured by low-pH comet assay, than more sedentary birds (General Linear Model *F* = 5.99, d.f. = 1,22, *P* = 0.025). Proportions of DNA damage were arcsine square root transformed, and activity counts (principal component 1; PC1) were square root transformed prior to analysis.

Using the low-pH comet assay, there was a higher proportion of damaged DNA in more active birds (Activity PC1) than in more sedentary birds (Fig. 2; GLM *F* = 5.99, d.f. = 1,22, *P* = 0.025). Activity was not significantly correlated with MDA, H_2_O_2_ comet assay or the high-pH comet assay.

None of the measures of oxidative damage were significantly related to one another, as follows: MDA and low-pH comet assay (ρ = −0.076, *n* = 20, *P* = 0.75); MDA and high-pH comet assay (ρ = −0.286, *n* = 22, *P* = 0.29); MDA and H_2_O_2_-treated comet assay (ρ = 0.176, *n* = 22, *P* = 0.43); low-pH and high-pH comet assays (ρ = 0.017, *n* = 19, *P* = 0.94); low-pH and H_2_O_2_-treated comet assays (ρ = −0.09, *n* = 19, *P* = 0.69); and high-pH and H_2_O_2_-treated comet assays (ρ = 0.31, *n* = 22, *P* = 0.16).

### Behavioural activity and body mass

Body mass measures were not linked with PCA scores of activity levels (*P* > 0.4 in all cases). Feeding duration during the behavioural trials was positively correlated with body mass at the end of the experiment (ρ = 0.50, *n* = 24, *P* = 0.012) and change in mass (ρ = 0.54, *n* = 24, *P* = 0.012) but not with mass at the start of the experiment (*P* > 0.9). Thus, budgerigars that spent more time eating were heavier compared with those that spent less time feeding after a short period without food.

### Plasma antioxidants and measures of oxidative damage

Mean values of antioxidant concentrations in budgerigar plasma (in micrograms per millilitre) were as follows: lutein, 33.78 ± 2.82; zeaxanthin, 31.83 ± 3.22; retinol, 0.69 ± 0.08; and α-tocopherol, 2.07 ± 0.83. There were no significant relationships between plasma antioxidant concentrations and any measure of oxidative damage (see Table 1). With the exception of lutein and zeaxanthin, concentrations of antioxidants were uncorrelated with each other (Table 1). Antioxidant concentrations were not correlated with natural variation in the frequencies of behaviours (*P* > 0.5 in all cases).

**Table 1:** Correlations between different plasma antioxidants (α-tocopherol, retinol, lutein and zeaxanthin) and measures of oxidative damage [malondialdehyde (MDA), high-pH, low-pH and H_2_O_2_-treated comet assays]

Parameter		Retinol	Lutein	Zeaxanthin	MDA	Comet high pH	Comet low pH	Comet H_2_O_2_
α-Tocopherol	rho	0.260	0.164	0.190	0.113	0.246	−0.075	−0.184
	*P*	0.30	0.50	0.44	0.66	0.32	0.77	0.47
Retinol	rho		0.267	0.257	0.319	−0.191	−0.043	−0.129
	*P*		0.28	0.30	0.21	0.46	0.87	0.62
Lutein	rho			0.926	−0.049	0.171	0.122	−0.013
	*P*			0.001	0.85	0.50	0.63	0.95
Zeaxanthin	rho				−0.106	0.093	0.084	−0.176
	*P*				0.68	0.71	0.74	0.49

Spearman's rho and probability (*P*) are shown (*n* = 17–19).

Birds that consumed on average a higher mass of seed during behavioural trials had higher circulating concentrations of lutein (ρ = 0.512, *n* = 19, *P* = 0.025) and zeaxanthin (ρ = 0.474, *n*= 19, *P* = 0.040), but not retinol (*P* > 0.4) or α-tocopherol (*P* > 0.3; see also Supplementary Results). Body mass at the start of the experiment was not related to plasma concentrations of antioxidants (*P* > 0.6 in all cases). However, mass gain during the experiment was positively related to plasma concentrations of retinol, zeaxanthin and α-tocopherol (GLM retinol, *F* = 13.78, d.f. = 1,17, *P* = 0.002; zeaxanthin, *F* = 8.04, d.f. = 1,18, *P* = 0.013; and α-tocopherol, *F* = 6.39, d.f. = 1,17, *P* = 0.024). Finally, budgerigars that were relatively heavy at the end of the experiment had significantly higher plasma concentrations of retinol (*F* = 11.77, d.f. = 1,18, *P* = 0.004), zeaxanthin (*F* = 5.52, d.f. = 1,18, *P* = 0.034) and α-tocopherol (*F* = 5.92, d.f. = 1,18, *P* = 0.029) than lighter individuals.

## Discussion

Our results showed that for budgerigars in standard pet cages fed *ad libitum* food, some measures of oxidative damage (but not others) were associated with activity and body mass. Interestingly, the H_2_O_2_-treated comet assay indicated that the DNA of birds that were heavy at the end of the experiment was most sensitive to future ROS damage. Although not statistically significant, the data indicate that this relationship between H_2_O_2_-treated comet assay and mass at the end of the experiment could be a result of both high mass at the start of the experiment and high mass gain while in the relatively small experimental cages. It should be noted that there is some controversy concerning exactly what the different comet assays reveal about DNA damage (Collins *et al.*, 2008; Speit *et al.*, 2009), but these assays are commonly used in human studies, particularly those testing the efficacy of nutritional supplements (reviewed by Hoelzl *et al.*, 2009). These heavier individuals did not seem to be deficient in dietary antioxidants, because they had significantly higher concentrations of retinol, zeaxanthin and α-tocopherol than lighter individuals. Although the long-term implications of DNA damage associated with higher body mass are unclear from the present study, increases in DNA damage can eventually lead to apoptosis (Monti *et al.*, 1992). Given that avian lymphocytes, the cells probed in the comet assay, are produced only early in development and circulate for lengthy periods (Glick, 1979), long-term DNA damage may induce a reduction in lymphocyte numbers and therefore leave an animal vulnerable to disease. This could have welfare and conservation implications for captive animals. These results indicate that an *ad libitum* diet, at least in association with captivity in a relatively small cage, may promote suboptimally high weight gain, with associated consequences for oxidative stress and health. The fact that the seed mix in our trial contained extra antioxidant-rich items that did not compensate for weight-related oxidative damage suggests that providing a restricted diet for captive animals to maintain their body mass (rather than gain mass) may be as important as providing appropriate nutrients.

We also wished to test whether unforced activity (i.e. the exercise that animals performed without human intervention) was associated with oxidative damage. Individuals that were more active in their cages had more damaged DNA, measured by low-pH comet assay, than more sedentary birds. Several studies have shown that exercise and activity are capable of increasing correlates of oxidative stress (e.g. Hartmann *et al.*, 1995; Aniagu *et al.*, 2006). However, many of the studies linking exercise and oxidative stress have enforced strenuous exercise on experimental subjects, and the extent to which ‘natural’ or ‘unforced’ behaviour is linked to oxidative damage is currently unclear (Tiidus, 1998). Previously, we have demonstrated that after strenuous flight activity, the MDA levels of budgerigars were significantly higher after a single training session than after 9 weeks of regular flight training. Moreover, at the start of that experiment, budgerigars that were relatively heavy for their skeletal size showed significantly higher post-exercise MDA levels than leaner individuals, but this relationship had disappeared after 9 weeks of exercise training (Larcombe *et al.*, 2010a). Both of these results were independent of the antioxidant content of the diet, suggesting a role for the endogenous antioxidant system in modulating responses to regular exercise or, conversely, to a sedentary life (reviewed by Urso and Clarkson, 2003). Our results in the present study therefore do not imply that exercise is harmful for captive birds. Exercise in sedentary individuals can cause oxidative damage, but regular training can improve the ability of the body to cope with strenuous exercise, as has been shown in mammals (Sen *et al.*, 1992; Oztasan *et al.*, 2004). In order to protect against the deleterious nature of oxidative stress, it seems likely that the exercise regimen of captive birds, especially long-lived species, such as parrots, should be considered where they are kept for conservation purposes. We suggest that even cage sizes that permit short flights and the stretching of wings might be insufficient to allow captive birds to obtain the benefits of exercise-mediated upregulation of antioxidant systems.

An important consideration in our results is that defining ‘exercise’ or ‘activity’ in unmanipulated captive animals is extremely difficult. In this trial, we recorded all the behaviours performed by the budgerigars and used a principal component analysis to reduce the complexity to two measures. Interestingly, only one of these measures seemed related to oxidative damage, i.e. the principal component explained by flight, walking and hopping. We might consider that these three behaviours represent the most ‘active’ of any behaviour we recorded, based on the idea that without the ability to perform strenuous exercise regularly, such active behaviours might promote oxidative damage. The results support this view, although it should be noted that other factors might underline this relationship. Physiological stress is known to promote oxidative stress and damage (Costantini *et al.*, 2011). In the present trial, we wished to assess the impact of captivity in small cages on exercise and oxidative damage, although it is possible that moving into smaller cages was stressful for the birds (Dickens *et al.*, 2009); captive-bred birds have been shown to have attenuated stress hormone responses compared with wild-bred birds (Cabezas *et al.*, 2013). Behaviour interpreted as ‘active’ might also reflect agitation associated with stress. In either case, where birds are required to be caged for conservation purposes, we recommend that large aviaries would allow both increased exercise and, potentially, a reduction in physiological stress.

Another notable aspect of our results is that different measures of oxidative damage and dietary antioxidant profile were uncorrelated with one another. This has also been found in studies on humans in relationship to dietary supplements (Hoelzl *et al.*, 2009) and in studies on birds (reviewed by Costantini and Verhulst, 2009). These results show that defining oxidative status is complex; absence of any effect on one measure of oxidative damage does not indicate a *de facto* absence of change in oxidative stress. Malondialdehyde is directly representative of levels of lipid peroxidation, one of the major types of oxidative damage. The finding that lipid peroxidation, which has been reported as being exacerbated by exercise (Vollaard *et al.*, 2005), was unrelated to natural activity levels in our study is potentially significant for our understanding of the mechanisms of oxidative damage. All of the results discussed above as oxidative stress relate to DNA damage measured by comet assay. Comet assay employs only DNA from lymphocytes, but the origins of plasma byproducts of lipid peroxidation, such as MDA, are unknown. Thus, there are probably tissue-specific products of oxidative stress. Plasma levels of antioxidants were unrelated to levels of oxidative damage, although both traits were associated with very high body mass in our budgerigars. It is possible that antioxidants in plasma were used up in countering oxidative stress prior to blood sampling or that important antioxidants were stored in tissues, rather than immediately used or circulated in plasma (Surai, 2002). Of course, other, unmeasured, antioxidants may also be valuable. Moreover, a range of antioxidants could act synergistically in limiting oxidative damage in this species (Ewen *et al.*, 2006). Although our analyses of MDA and DNA damage cannot be used as an assessment of total oxidative stress (Dotan *et al.*, 2004; Larcombe *et al.*, 2008), we suggest that measuring products of oxidative damage is a more effective measure of oxidative status than measuring antioxidant capacity, because the potential of molecules to act as antioxidants *in vitro* does not necessitate that this will be their role *in vivo* (Costantini and Verhulst, 2009). Moreover, we have shown that heavier budgerigars also had higher concentrations of plasma antioxidants than lighter individuals. Thus, antioxidant status is not necessarily an index of ‘health’ or ability to withstand oxidative damage. In our study, simply using antioxidant wealth as a measure of oxidative status, as in other studies (reviewed by Costantini and Møller, 2008), would have led to misleading conclusions.

In the present study, we found that active behaviour was linked with oxidative damage, in that more active budgerigars had more single-stranded breaks in their DNA than more sedentary individuals. Individuals varied in their foraging behaviour following a short period without food, and this was related to their body mass. Although causality needs to be determined, it suggests that appetite and thus overeating varies between individuals kept in standardized conditions. We also demonstrated, for the first time in captive birds, that mass gain and body mass are linked to DNA damage and sensitivity of DNA to future ROS attack. This study opens the door to further work on the extent to which exercise and feeding regimens can alter oxidative profile, and thus fitness-related traits. Moreover, our data gathered in standardized conditions have important implications for understanding the mechanisms underlying the curtailed lifespan and fecundity common in many Pscittaciformes kept for conservation purposes.

## Supplementary material

Supplementary material is available at *Conservation Physiology* online.

## Funding

S.D.L. was funded by a BBSRC Industrial CASE studentship and K.E.A. by a Royal Society University Research Fellowship. Other funding was provided by WALTHAM^®^ Centre for Pet Nutrition.

## Supplementary Material

Supplementary Data

## References

[COV045C1] Aniagu SO, Day N, Chipman JK, Taylor EW, Butler PJ, Winter MJ (2006) Does exhaustive exercise result in oxidative stress and associated DNA damage in the chub (*Leuciscus cephalus*)? Environ Mol Mutagen 47: 616–623. 16878316 10.1002/em.20247

[COV045C2] Arnold KE, Herborn KA, Adam A, Alexander L (2015) Individual variation in the oxidative costs of personality traits. Funct Ecol 29: 522–530.

[COV045C3] Aroor AR, DeMarco VG (2014) Oxidative stress and obesity: the chicken or the egg? Diabetes 63: 2216–2218. 24962921 10.2337/db14-0424

[COV045C4] Bonisoli-Alquati A, Voris A, Mousseau TA, Møller AP, Saino N, Wyatt MD (2010) DNA damage in barn swallows (*Hirundo rustica*) from the Chernobyl region detected by use of the comet assay. Comp Biochem Physiol C Toxicol Pharmacol 151: 271–277. 19941973 10.1016/j.cbpc.2009.11.006

[COV045C5] Brockway BF (1964) Social influences on reproductive physiology and ethology of budgerigars (*Melopsittacus undulatus*). Anim Behav 12: 493–501.

[COV045C6] Brue RN (1994) Chapter 3: Nutrition. In Ritchie BW, Harrison GJ, Harrison LR, eds, Avian Medicine Principles and Application. Wingers Publishing Inc, Lake Worth, FL, USA, pp 63–95.

[COV045C7] Cabezas S, Carrete M, Tella JL, Marchant TA, Bortolotti GR (2013) Differences in acute stress responses between wild-caught and captive-bred birds: a physiological mechanism contributing to current avian invasions? Biol Invasions 15: 521–527.

[COV045C8] Clubb R, Rowcliffe M, Lee P, Mar KU, Moss C, Mason GJ (2009) Fecundity and population viability in female zoo elephants: problems and possible solutions. Anim Welfare 18: 237–247.

[COV045C200] Collins AR, Oscoz AA, Brunborg G, Gaivão I, Giovannelli L, Kruszewski M, Smith CC, Štětina R (2008) The comet assay: topical issues. Mutagenesis 23: 143–151. 18283046 10.1093/mutage/gem051

[COV045C9] Costantini D, Møller AP (2008) Carotenoids are minor antioxidants for birds. Funct Ecol 22: 367–370.

[COV045C10] Costantini D, Verhulst S (2009) Does high antioxidant capacity indicate low oxidative stress? Funct Ecol 23: 506–509.

[COV045C11] Costantini D, Cardinale M, Carere C (2007) Oxidative damage and anti-oxidant capacity in two migratory bird species at a stop-over site. Comp Biochem Physiol C Toxicol Pharmacol 144: 363–371. 17218158 10.1016/j.cbpc.2006.11.005

[COV045C12] Costantini D, Marasco V, Møller AP (2011) A meta-analysis of glucocorticoids as modulators of oxidative stress in vertebrates. J Comp Physiol B 181: 447–456. 21416253 10.1007/s00360-011-0566-2

[COV045C13] Costantini D, Monaghan P, Metcalfe NB (2013) Loss of integration is associated with reduced resistance to oxidative stress. J Exp Biol 216: 2213–2220. 23470664 10.1242/jeb.083154

[COV045C14] Dickens MJ, Earle KA, Romero LM (2009) Initial transference of wild birds to captivity alters stress physiology. Gen Comp Endocrinol 160: 76–83. 19026651 10.1016/j.ygcen.2008.10.023

[COV045C15] Diplock AT (1994) Antioxidants and disease prevention. Mol Aspects Med 15: 293–376. 7845187 10.1016/0098-2997(94)90005-1

[COV045C16] Dotan Y, Lichtenberg D, Pinchuk I (2004) Lipid peroxidation cannot be used as a universal criterion of oxidative stress. Prog Lipid Res 43: 200–227. 15003395 10.1016/j.plipres.2003.10.001

[COV045C17] Earnhardt J, Velez-Valentin J, Valentin R, Long S, Lynch C, Schowe K (2014) The Puerto Rican parrot reintroduction program: sustainable management of the aviary population. Zoo Biol 33: 89–98. 24395187 10.1002/zoo.21109

[COV045C18] Ewen JG, Thorogood R, Karadas F, Pappas AC, Surai PF (2006) Influences of carotenoid supplementation on the integrated antioxidant system of a free living endangered passerine, the hihi (*Notiomystis cincta*). Comp Biochem Physiol A Mol Integr Physiol 143: 149–154. 16406271 10.1016/j.cbpa.2005.11.006

[COV045C19] Fidgett AL, Gardner L (2014) Advancing avian nutrition through best feeding practice. Int Zoo Yearb 48: 116–127.

[COV045C20] Forshaw J (2010) Parrots of the World. Princeton University Press, Princeton, USA.

[COV045C21] Furukawa S, Fujita T, Shimabukuro M, Iwaki M, Yamada Y, Nakajima Y, Nakayama O, Makishima M, Matsuda M, Shimomura I (2004) Increased oxidative stress in obesity and its impact on metabolic syndrome. J Clin Invest 114: 1752–1761. 15599400 10.1172/JCI21625 PMC535065

[COV045C22] German AJ, Holden SL, Wiseman-Orr ML, Reid J, Nolan AM, Biourge V, Morris PJ, Scott EM (2012) Quality of life is reduced in obese dogs but improves after successful weight loss. Vet J 192: 428–434. 22075257 10.1016/j.tvjl.2011.09.015

[COV045C23] Glick B (1979) Avian immune system. Avian Dis 23: 282–289.

[COV045C24] Harper EJ (1998) Hematology values in a colony of budgerigars (*Melopsittacus undulatus*) and changes associated with aging. J Nutr 128: 2639–2640. 10.1093/jn/128.12.2639S 9868226

[COV045C25] Hartmann A, Niess AM, Grünert-Fuchs M, Poch B, Speit G (1995) Vitamin E prevents exercise-induced DNA damage. Mutat Res 346: 195–202. 7753111 10.1016/0165-7992(95)90035-7

[COV045C26] Hawkins P, Morton DB, Cameron D, Cuthill I, Francis R, Freire R, Gosler A, Healy S, Hudson A, Inglis I et al (2001) Laboratory birds: refinements in husbandry and procedures. Lab Anim 35: S1–S163.

[COV045C27] Hegseth MN, Regoli F, Gorbi S, Bocchetti R, Gabrielsen GW, Camus L (2011) Lysosomal and lipid-associated parameters in the livers of three species of Arctic seabird chicks: species differences and relationships with contaminant levels. Mar Pollut Bull 62: 1652–1660. 21724205 10.1016/j.marpolbul.2011.06.011

[COV045C28] Hemmings N, West M, Birkhead TR (2012) Causes of hatching failure in endangered birds. Biol Lett 8: 964–967. 22977070 10.1098/rsbl.2012.0655 PMC3497133

[COV045C29] Hoelzl C, Knasmüller S, Misík M, Collins A, Dusinská M, Nersesyan A (2009) Use of single cell gel electrophoresis assays for the detection of DNA-protective effects of dietary factors in humans: recent results and trends. Mutat Res 681: 68–79. 18755290 10.1016/j.mrrev.2008.07.004

[COV045C30] Houston D, McInnes K, Elliott G, Eason D, Moorhouse R, Cockrem J (2007) The use of a nutritional supplement to improve egg production in the endangered kakapo. Biol Conserv 138: 248–255.

[COV045C31] Kalmar ID, Janssens GPJ, Moons CPH (2010) Guidelines and ethical considerations for housing and management of psittacine birds used in research. ILAR J 51: 409–423. 21131717 10.1093/ilar.51.4.409

[COV045C32] Knight JA (1998) Free radicals: their history and current status in aging and disease. Ann Clin Lab Sci 28: 331–346. 9846200

[COV045C33] Kolosova NG, Shcheglova TV, Sergeeva SV, Loskutova LV (2006) Long-term antioxidant supplementation attenuates oxidative stress markers and cognitive deficits in senescent-accelerated OXYS rats. Neurobiol Aging 27: 1289–1297. 16246464 10.1016/j.neurobiolaging.2005.07.022

[COV045C34] Koutsos EA, Matson KD, Klasing KC (2001) Nutrition of birds in the order Psittaciformes: a review. J Avian Med Surg 15: 257–275.

[COV045C35] Larcombe SD, Tregaskes C, Coffey JS, Stevenson AE, Alexander L, Arnold KE (2008) The effects of short-term antioxidant supplementation on oxidative stress and flight performance in adult budgerigars *Melopsittacus undulatus*. J Exp Biol 211: 2859–2864. 18723545 10.1242/jeb.017970

[COV045C36] Larcombe SD, Coffey JS, Bann D, Alexander L, Arnold KE (2010a) Impacts of dietary antioxidants and flight training on post-exercise oxidative damage in adult parrots. Comp Biochem Physiol B Biochem Mol Biol 155: 49–53. 19800412 10.1016/j.cbpb.2009.09.009

[COV045C37] Larcombe SD, Mullen W, Alexander L, Arnold KE (2010b) Dietary antioxidants, lipid peroxidation and plumage colouration in nestling blue tits *Cyanistes caeruleus*. Naturwissenschaften 97: 903–913. 20838757 10.1007/s00114-010-0708-5

[COV045C38] Metcalfe NB, Monaghan P (2013) Does reproduction cause oxidative stress? An open question. Trends Ecol Evol 28: 347–350. 23485157 10.1016/j.tree.2013.01.015

[COV045C39] Monaghan P, Metcalfe NB, Torres R (2009) Oxidative stress as a mediator of life history trade-offs: mechanisms, measurements and interpretation. Ecol Lett 12: 75–92. 19016828 10.1111/j.1461-0248.2008.01258.x

[COV045C40] Monti D, Troiano L, Tropea F, Grassilli E, Cossarizza A, Barozzi D, Pelloni MC, Tamassia MG, Bellomo G, Franceschi C (1992) Apoptosis—programmed cell-death: a role in the aging process? Am J Clin Nutr 55: S1208–S1214. 10.1093/ajcn/55.6.1208S 1590258

[COV045C300] Murphy MP, Holmgren A, Larsson N-G, Halliwell B, Chang CJ, Kalyanaraman B, Rhee SG, Thornalley PJ, Partridge L, Gems D et al (2011) Unraveling the biological roles of reactive oxygen species. Cell Metab 13: 361–366. 21459321 10.1016/j.cmet.2011.03.010 PMC4445605

[COV045C41] OIE (2014) World Organisation for Animal Health: terrestrial animal health code (vol. 1) http://www.oie.int/en/international-standard-setting/terrestrial-code/access-online/.

[COV045C42] Oztasan N, Taysi S, Gumustekin K, Altinkaynak K, Aktas O, Timur H, Siktar E, Keles S, Akar S, Akcay F et al (2004) Endurance training attenuates exercise-induced oxidative stress in erythrocytes in rat. Eur J Appl Physiol 91: 622–627. 14685869 10.1007/s00421-003-1029-6

[COV045C43] Selman C, Blount JD, Nussey DH, Speakman JR (2012) Oxidative damage, ageing, and life-history evolution: where now? Trends Ecol Evol 27: 570–577. 22789512 10.1016/j.tree.2012.06.006

[COV045C44] Sen CK, Marin E, Kretzschmar M, Hänninen O (1992) Skeletal-muscle and liver glutathione homeostasis in response to training, exercise, and immobilization. J Appl Physiol 73: 1265–1272. 1360001 10.1152/jappl.1992.73.4.1265

[COV045C45] Sepp T, Karu U, Blount JD, Sild E, Männiste M, Hõrak P (2012) Coccidian infection causes oxidative damage in greenfinches. PLoS ONE 7: e36495. 22615772 10.1371/journal.pone.0036495 PMC3352913

[COV045C46] Snyder NFR, Derrickson SR, Beissinger SR, Wiley JW, Smith TB, Toone WD, Miller B (1996) Limitations of captive breeding in endangered species recovery. Conserv Biol 10: 338–348.

[COV045C400] Speit G, Vasquez M, Hartmann A (2009) The comet assay as an indicator test for germ cell genotoxicity. Mutat Res Rev Mutat Res 681: 3–12. 10.1016/j.mrrev.2008.03.005 18462987

[COV045C47] Surai PF (2002) Natural Antioxidants in Avian Nutrition and Reproduction. Nottingham University Press, Nottingham, UK.

[COV045C48] Tice RR, Aqurell E, Anderson D, Burlinson B, Hartmann A, Kobayashi H, Miyamae Y, Rojas E, Ryu JC, Sasaki YF (2000) Single cell gel/comet assay: guidelines for in vitro and in vivo genetic toxicology testing. Environ Mol Mutagen 3: 206–221. 10.1002/(sici)1098-2280(2000)35:3<206::aid-em8>3.0.co;2-j 10737956

[COV045C49] Tiidus PM (1998) Radical species in inflammation and overtraining. Can J Physiol Pharmacol 76: 533–538. 9839079 10.1139/cjpp-76-5-533

[COV045C50] Urso ML, Clarkson PM (2003) Oxidative stress, exercise, and antioxidant supplementation. Toxicology 189: 41–54. 12821281 10.1016/s0300-483x(03)00151-3

[COV045C51] Vollaard NBJ, Shearman JP, Cooper CE (2005) Exercise-induced oxidative stress myths, realities and physiological relevance. Sports Med 35: 1045–1062. 16336008 10.2165/00007256-200535120-00004

[COV045C52] White TH Jr, Collar NJ, Moorhouse RJ, Sanz V, Stolen ED, Brightsmith DJ (2012) Psittacine reintroductions: common denominators of success. Biol Conserv 148: 106–115.

[COV045C53] Woodall AA, Britton G, Jackson MJ (1996) Dietary supplementation with carotenoids: effects on α-tocopherol levels and susceptibility of tissues to oxidative stress. Br J Nutr 76: 307–317. 8813904 10.1079/bjn19960034

[COV045C54] Young AM, Hobson EA, Lackey LB, Wright TF (2012) Survival on the ark: life-history trends in captive parrots. Anim Conserv 15: 28–43. 22389582 10.1111/j.1469-1795.2011.00477.x PMC3289156

[COV045C55] Young IS, Trimble ER (1991) Measurement of malondialdehyde in plasma by high performance liquid chromatography with fluorometric detection. Ann Clin Biochem 28: 504–508. 1958055 10.1177/000456329102800514

