# Supplementary material for: Oxidative stress, activity behaviour and body mass in captive parrots
Source: Conserv Physiol. 2015 Oct 20;3(1):cov045. doi: 10.1093/conphys/cov045 (PMC4778434; doi:10.1093/conphys/cov045)
Supplement: Supplementary Data [file cov045supp.zip › cov045supp.docx]

**Supplementary Material**

**Oxidative stress, activity behaviour and body mass in captive parrots**

S. D. Larcombe^1^, C. A. Tregaskes^2, 3^, J. Coffey^2^, A. Stevenson^2^, L.G. Alexander^2, 4^, K. E. Arnold^5^

**METHODS**

*Variation in behaviour*

We calculated ‘Maintenance’ behaviour as frequency of preening, stretching and bill wiping. ‘Active’ behaviour was scored as the frequency of flying, walking, climbing, turning (where birds would jump round on the spot), and hopping. Finally ‘Feeding’ was calculated as the combined frequency of eating or drinking. Birds exhibited a broad range of behaviours, most often engaging in Active (excluding climbing), and Feeding behaviours (Fig 1).

*Food choice and intake*

At 8.00am on days 24-26 of the experiment, feeding dishes were removed from each cage for a period of 2 hours to standardise hunger, and cages were cleaned. Next, pairs were separated with the female on the left of the cage in all cases. Individual budgerigars were presented with a food bowl containing a prepared 10g food sample containing identical proportions of each seed and Nutrivit®. The video camera in front of each cage was switched on during the food choice trial to record behaviour without the confounding effects of social interactions, though the birds were not acoustically isolated. The dish and tray, along with any spilled seed, were removed after 2 hours and the camera was turned off. The contents of the dish and cage floor were then sorted and weighed, and the number of Nutrivit® particles counted. Food choice was defined as the number of Nutrivit® pieces eaten, and the total mass of food eaten in the two hour period

*MDA analysis*

The MDA method was based on that of Young and Trimble (1991)([Young *et al.*, 1991](#_ENREF_3)) see ([Larcombe *et al.*, 2008](#_ENREF_1)) for full details. Briefly, thiobarbituric acid (0.044M, 100µl) and phosphoric acid (1.22M, 100µl) were mixed together and added to 50µl of plasma (per bird) in a test tube. An inert atmosphere was created by applying a nitrogen blanket, and the test tubes were sealed and vortexed prior to heating (60 min, 70-75^o^C). Samples were cooled in water, then 200µl was transferred to a centrifuge tube containing sodium hydroxide (1M, 100µl). Methanol (500µl) was added and mixed. Samples were centrifuged (10 min, 12000 g) and the supernatant analysed on a Summit HPLC system (Dionex, Idstein, Germany) using Chromeleon software (Dionex). An Acclaim 120 C18 5( 4.6 x 250 mm column (Dionex) and guard were used with fluorescence detection (excitation 532nm and emission 553nm). The mobile phase was isocratic, 40:60 methanol:phosphate buffer (40mM, pH 6.5), with a flow rate of 1ml/min, and a run time of 7 min. Samples were assayed against a standard of malonaldehyde bis (dimethyl acetal), (Sigma Aldrich, Poole, UK) that was simultaneously taken through the same procedure.

*Comet assay*

For each bird all three different treatment regimes were used: carrying out the electrophoresis at two different pH values (ie. High (alkaline) and lower) and in addition we treated cells with hydrogen peroxide at a lower pH.  High pH reveals both DNA single strand breaks and alkali sensitive sites, whereas the lower pH only reveals DNA single strand breaks. H2O2 is believed only to cause breaks, but not at alkali sensitive sites, and is suggested to indicate the susceptibility of DNA to oxidative damage.   The comet assay involved slow spin preparation of avian lymphocytes, treatment of cells with hydrogen peroxide (H2O2_)_ and embedding in agarose on slides, following the procedure in Tice et al. (2000)([Tice *et al.*, 2000](#_ENREF_2)). Next, we performed electrophoresis at Low pH (0.03M NaOH) to reveal DNA strand breaks and electrophoresis at high pH (0.3M NaOH) which also converts alkali-labile sites into single strand breaks. Slides were made and analysed on the same day as blood sampling. First slides were pre-prepared by pipetting 2 spots of 85ul of HMAgarose at 95^0^C on to each of 4 frosted microscope slide per bird and covered with glass coverslip. These were left to set at 4^o^C for 10 minutes. Next, bird blood was centrifuged at 50g for 5 minutes. The supernatant containing cells was removed taking care not to disturb the ball of red cells. 50μl of supernatant was mixed with 50μl of trypan blue and cells were counted using haemocytometer. Cells were diluted using cold PBS to a concentration of 4x10^4^ cells/ml. We made 5ml for each bird (2x10^5^ cells). We pipetted 0.5ml of 100μM H_2_O_2_ into 6ml polystyrene tubes (four per bird). The same was done for the untreated control (0.5ml of PBS in 6ml tubes - four per bird). 0.5ml of cells were added to each tube and incubated on ice for 5 minutes. 4ml of cold PBS was added to each tube, which was then centrifuged at 200g for 5 minutes. The coverslips were removed from the HMP agarose layers. The cells were combined with 85ul LMAgarose at 37^o^C and pipetted immediately on to the HMP agarose. The cover slip was replaced immediately and we left it at 4^o^C to set for 10 minutes. Once the slides had set, the cover slips were removed and the slides immersed in chilled lysis solution. At this point the slides were divided dependant on electrophoresis buffer and put into different containers at 4^o^C for a minimum of 65 minutes. The lysis solution was poured off and replaced with the appropriate electrophoresis solution (0.03M or 0.3M NaOH), and incubated in a fridge for 30 minutes. Two slides per bird per alkalinity (high or low), plus controls, were prepared for electrophoresis. Slides were transferred to the electrophoresis tank, with the slide label (left end of the slide) toward the anode (black). Fresh alkali electrophoresis solution (0.03M or 0.3M NaOH) was poured over to cover the slides. We then set the power supply to constant voltage at 0.8v/cm and performed electrophoresis for 30 minutes. Excess solution was poured off and slides were immersed in fresh neutralising solution three times for 5 minutes at 4^o^C. After the final wash, the slides were stored in a copeland jar with a small volume of neutralising buffer in the bottom to keep them moist, until they were scored. Excess moisture was removed from each slide and 50ul of SYBR Gold was applied to the gel. The slide was viewed by epifluoresence microscopy using an Olympus BX-51(Olympus Optical Co., Tokyo, Japan) with a 460nm UV filter for SYBR Green. Komet software (v.6, Kinetics Imaging, Nottingham, UK) was used for image analysis on 100 randomly selected cells for each bird and pH treatment. Cells were scored according to % DNA in the comet head, as a measure of DNA intactness. The mean intactness was calculated across the 100 cells per slide and across the two slides per treatment per bird. There was high repeatability > 80% across the two slides per treatment per bird.

RESULTS

*Variation in behaviour*

Behaviour – see Figure S1.

*Food choice and intake*

There were no significant relationships between the different behaviour scores and amount of seed or Nutrivit® eaten (p > 0.5 in all cases) or body mass (p > 0.15 in all cases). The mean feeding frequency was unrelated to average amount of seed or Nutrivit® eaten (p > 0.26). Plasma levels of antioxidants were unrelated to Nutrivit® uptake (p > 0.05 in all cases).

Supplementary Figure 1: Mean (± S.E.) frequencies per hour of different behaviours for all birds. The four behavioural categories analysed were: Active behaviours (grey), Flying (white), Maintenance behaviours (cross hatching) and Feeding behaviours (dots).

References

**Larcombe SD, Tregaskes C, S.Coffey J, Stevenson AE, Alexander L, Arnold KE** (2008) The effects of short-term antioxidant supplementation on oxidative stress and flight performance in adult budgerigars *melopsittacus undulatus*. *Journal of Experimental Biology* 211: 2859-2864

**Tice RR, Aqurell E, Anderson D, Burlinson B, Hartmann A, Kobayashi H, Miyamae Y, Rojas E, Ryu JC, Sasaki YF** (2000) Single cell gel/comet assay: Guidelines for in vitro and in vivo genetic toxicology testing. *Environ Mol Mutagen* 3: 206-221

**Young IS, Trimble ER** (1991) Measurement of malondialdehyde in plasma by high-performance liquid-chromatography with fluorometric detection. *Annals of Clinical Biochemistry* 28: 504-508
